# Synaptic Wnt/GSK3*β* Signaling Hub in Autism

**DOI:** 10.1155/2016/9603751

**Published:** 2016-01-10

**Authors:** Mario O. Caracci, Miguel E. Ávila, Giancarlo V. De Ferrari

**Affiliations:** ^1^Center for Biomedical Research, Faculty of Biological Sciences and Faculty of Medicine, Universidad Andres Bello, P.O. Box 8370134, Santiago, Chile; ^2^FONDAP Center for Genome Regulation, Santiago, Chile

## Abstract

Hundreds of genes have been associated with autism spectrum disorders (ASDs) and the interaction of weak and* de novo* variants derive from distinct autistic phenotypes thus making up the “spectrum.” The convergence of these variants in networks of genes associated with synaptic function warrants the study of cell signaling pathways involved in the regulation of the synapse. The Wnt/*β*-catenin signaling pathway plays a central role in the development and regulation of the central nervous system and several genes belonging to the cascade have been genetically associated with ASDs. In the present paper, we review basic information regarding the role of Wnt/*β*-catenin signaling in excitatory/inhibitory balance (E/I balance) through the regulation of pre- and postsynaptic compartments. Furthermore, we integrate information supporting the role of the glycogen synthase kinase 3*β* (GSK3*β*) in the onset/development of ASDs through direct modulation of Wnt/*β*-catenin signaling. Finally, given GSK3*β* activity as key modulator of synaptic plasticity, we explore the potential of this kinase as a therapeutic target for ASD.

## 1. Introduction

Autism spectrum disorders (ASDs) are highly heterogeneous, pervasive developmental disorders characterized by impaired social communication skills, repetitive behaviors, and a restricted range of interests [1]. The wide range of phenotypical traits regarding comorbidities and various degrees of cognitive and language impairments makes up the “spectrum” and adds complexity to the determination of genetic markers associated with a distinct phenotype [2]. ASDs have a strong genetic component as ascertained by a 90% concordance among monozygotic twins [3]. Significant advancements have been made in identifying molecular mechanisms involved in ASDs by studying disorders with Mendelian inheritance patterns such as Tuberous Sclerosis complex (*TSC1 *and* TSC2*), Rett syndrome (*MECP2*), Fragile X syndrome (FXS; which results from mutated Fragile X mental retardation-1,* FMR1*), and Cowden syndrome (*PTEN*), but, altogether, these disorders do not account for more than 10% of cases [4]. In the last few years, efforts have focused on understanding the genetic contribution of single nucleotide variants (SNVs) and copy number variants (CNVs) in ASD [5, 6]. While genome wide association studies (GWAS) have identified over 100 genes associated with ASDs, most of the variants identified have a weak effect suggesting a greater contribution for rare variants [7]. Rare variants and* de novo* occurring SNVs and CNVs have a larger contribution to the onset of ASD [6]. Indeed,* de novo* CNVs are significantly enriched in individuals affected with the disorder and it is estimated that 8% of cases that carry these variants are likely to be pathogenic [8, 9]. On the other hand, 9% of* de novo* SNVs in affected individuals are disruptive or frameshift mutations that generate nonconserved amino acid changes such as premature stop codons or alternative splice sites ultimately affecting the normal biological function of the resulting protein [10, 11]. Overall, it is estimated that these deleterious* de novo* variants affect ASD susceptibility in 10–15% of probands [10, 11]. Nevertheless, exomic data suggests that no single gene could account for more than 1% of ASD cases, which makes it difficult to target a single protein to treat autistic behaviors. More recently, the integration of these genes into functional networks has allowed the identification of specific molecular pathways that could be disrupted in ASD [12, 13]. In this regard, recent exome sequencing studies in family trios identified that 39% of the more disruptive* de novo* mutations are part of an interconnected network of chromatin remodeling, synaptic plasticity, and Wnt/*β*-catenin signaling genes [13–15].

Through the analysis of biochemical and pharmacological data, animal models of the disease, and genetic association studies, we predicted earlier that the onset/development of ASDs might involve the additive effect of genetic variants within Wnt/*β*-catenin signaling components and/or genes coding for molecules that modulate its functional activity [16], and such hypothesis has received considerable attention recently [6, 17, 18]. Wnts are lipid modified secreted glycoproteins that signal through three major pathways: the Planar Cell Polarity (PCP), Wnt/Ca^+2^, and the canonical Wnt/*β*-catenin signaling pathway [19]. Wnt/*β*-catenin signaling is the most well understood cascade and it starts via binding of the Wnt ligand to cell membrane receptors Frizzled (*FZD*), belonging to the 7-transmembrane domains family of proteins and to members of the low density lipoprotein receptor related proteins 5 and 6 (*LRP5*/*6*), which act as coreceptors [20]. Wnt binding to its membrane receptor activates intracellular signaling leading to the dissociation of *β*-catenin from the degradation complex consisting of Axin and adenomatous polyposis coli (APC) scaffolds [21], and the serine-threonine kinases casein kinase 1 (CK1) and glycogen synthase kinase 3*β* (GSK3*β*) [22]. As a net result, *β*-catenin accumulates in the cytosol and translocates to the nucleus where it interacts with T-cell factor/lymphoid enhancing factor (TCF/LEF) transcription factors to activate transcription of target genes [23]. Conversely, in the absence of a Wnt ligand, Axin and APC facilitate CK1 and GSK3*β* sequential phosphorylation of *β*-catenin [22] targeting the protein for ubiquitination by the *β*-transducing repeat-containing protein (*β*-TrCP) and subsequent proteasome degradation [24].

It is interesting to note that the tumor suppressor complex formed by TSC1 and TSC2 interacts with the *β*-catenin degradation complex and thus modulates the action of Wnt signaling [25, 26]. Other genetic elements associated with ASDs are the canonical Wnt2 ligand [27], the hepatocyte growth factor receptor (MET) [28, 29], which is a target gene of Wnt/*β*-catenin signaling [30], and several genes encoding for cadherins, including* CDH5*,* CDH8*,* CDH9*,* CDH10*,* CDH13*,* CDH15*,* PCDH10*,* PCDH19,* and* PCDHb4* [31], some of which may interact with *β*-catenin in cell-cell adhesion complexes. More recently, the chromo-helicase domain protein 8 (CHD8) [13, 14, 32], which inhibits *β*-catenin through direct binding [33], and DYRK1A that modulates Wnt signaling through interaction with the p120 catenin [34] have been found to be associated with ASDs. Interestingly, these genes harbor recurrent disruptive mutations and display a high correlation with head size abnormalities [14], which is a feature commonly observed during the first 2-3 years of life of an ASD individual [35]. Finally, rare* de novo* genetic variants in the *β*-catenin (*CTNNB1*) gene itself have been implicated in severe intellectual disability [36]. Therefore, the convergence of genetic markers in synaptic components opens a therapeutic window that aims not only to correct developmental brain abnormalities, but also to compensate the inherent plasticity through modulation of the highly dynamic synapse. In the present paper, we review current knowledge of synaptic transmission leading to excitatory and inhibitory (E/I) imbalance commonly seen in ASD and how this phenomenon relates to dysfunction of the Wnt/*β*-catenin pathway. Furthermore, we trace functional defects to GSK3*β* activity and explore its pharmacological regulation as a potential therapeutic target for ASD, particularly in relation to synaptic plasticity.

## 2. Wnt/***β***-Catenin Signaling and Synaptic Transmission Defects in ASDs

The inherent ability of the brain to process information is accomplished by a highly sophisticated network that allows long-distance communication between cells and which is largely based on the E/I balance from neuronal connections. Genetic, functional, and structural information suggests that the E/I balance may underlie the symptomatology of ASDs [37–39]. This idea has been examined through optogenetic methods in the medial prefrontal cortex of mice, and it was found that the elevation, but not the reduction, of cellular E/I balance (i.e., increase in excitatory transmission) induced cellular defects in information processing, leading to behavioral and social deficits [39]. E/I balance anomalies have similarly been observed in several ASD animal models, including the neuroligin 3 (*NLGN3*) mutant mice, and the models for Rett, Fragile X, and Angelman syndromes (Rev. in [40]). In humans, one of the most relevant evidence associating the E/I balance with ASDs is its high comorbidity with epilepsy (30% comorbidity with ASDs) [41, 42]. Epileptic activity can be triggered by blocking synaptic inhibitory transmission or by activating excitatory transmission linking the E/I imbalance in the establishment of epileptiform seizures [43].

**Figure 1:**
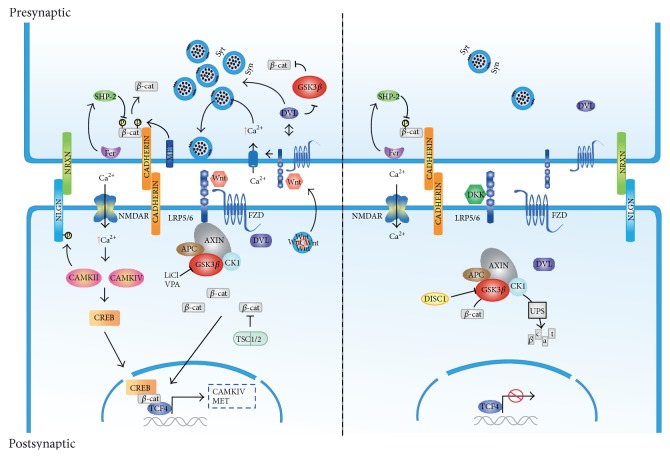
Wnt/*β*-catenin signaling in ASDs. Wnt binding to FZD-LRP5/6 complex receptor at the membrane recruits the destruction complex and inhibits GSK3*β* activity thus stabilizing *β*-catenin in the cytoplasm and nucleus. Activation of the Wnt/*β*-catenin pathway facilitates synaptic plasticity through the activation of voltage gated ion channels that allows activation of CAMK and CREB mediated transcription. Mutations in TSC associated with ASD prevent *β*-catenin degradation which results in a gain of function of the Wnt pathway. In the presynaptic terminal cadherin mediated cell adhesion between synapses is weakened by phosphorylation of *β*-catenin and synaptic vesicle clustering is enhanced through DVL1. Clustering is also dependent on NLGN/NRXN cell adhesion complexes. Both lithium (LiCl) and VPA activate Wnt/*β*-catenin signaling through inhibition of GSK3*β* activity. Conversely, in the absence of a Wnt ligand, activated GSK3*β* targets *β*-catenin for proteosome-mediated degradation. Mutations associated with DISC1 fail to inhibit GSK3*β* and thus activate Wnt/*β*-catenin pathway. In the presynaptic side Wnt signaling buffering of synaptic vesicles is inhibited and adherens junctions mediated by cadherins are strengthened.

Wnt signaling has been widely acknowledged during patterning, development, and maturation of functional synapses within the CNS [16, 44–48]. Wnt1, Wnt3a, Wnt7a, and Wnt8 are ligands known to activate Wnt/*β*-catenin signaling and are involved in brain development and synaptogenesis [49–51]. Wnt7a and Wnt8a have also been shown to regulate excitatory synaptic formation [45, 52]. Furthermore, a recent study suggests that LRP6, Wnt/*β*-catenin signaling coreceptor, is critical for the development of functional synapses* in vivo* [52], which further supports the involvement of Wnt/*β*-catenin signaling in synaptic development. Interestingly tetanic stimulation induces the release of the Wnt3a ligand from the postsynaptic terminal [53]. We demonstrated later that treatment with purified Wnt3a protein of cultured hippocampal neurons enhanced a fast influx of Ca^2+^ in the presynaptic terminal and enhanced mEPSC frequency at the postsynaptic terminal, in an LRP6-dependent mechanism [54]. Hence, the data suggests a prominent role for Wnt/*β*-catenin signaling in the regulation of excitatory synaptic transmission in pre- and postsynaptic compartments, thus ascribing a role for the signaling cascade in E/I balance regulation (Figure 1).

## 3. ASDs and Wnt Signaling at the Presynaptic Terminal 

At the presynaptic region, canonical Wnt signaling has a major role in clustering and recycling of synaptic vesicles (SVs). Conditioned media containing Wnt7a, and to a lesser extent Wnt3a, were found to enhance SVs recycling in primary cultures of rat hippocampal neurons [55]. Similarly, loss of Wnt7a function inhibits SVs clustering, an effect that is mimicked by loss of function of Dishevelled 1 (*DVL1*) signaling downstream of Wnt ligands [47]. Interestingly, Dvl1 knockout mice exhibit social interaction and sensorimotor abnormalities [56]. Moreover, the Wnt7a/Dvl1 double mutant mice show defects in spine morphogenesis and excitatory synaptic neurotransmission [45], which parallels behavioral abnormalities with a disrupted presynaptic assembly and E/I balance, as it is likely observed in ASDs.

Wnt/*β*-catenin signaling also seems to trigger neurotransmitter release and SV trafficking by modulating the function of SVs-associated phosphoproteins, including membrane-trafficking proteins such as synapsin and synaptotagmin. While all three members of the synapsin (SYN) gene family (*SYN1-3*) [57] have been associated with ASDs [58–60], it has been shown that canonical Wnt ligands such as Wnt7a and Wnt3a enhance the clustering [61] and phosphorylation [54] of Syn1 at the synaptic button prior to neurotransmitter release. Likewise,* SYN2* is predicted as a Wnt/*β*-catenin target gene [62] and is upregulated as a consequence of enhanced Wnt signaling activity in hippocampal neurons from APC conditional knockout mice that has impaired learning and memory and that displays ASD-like behaviors [63]. Finally, it was shown that the Wnt signaling component Dvl1 is involved in neurotransmitters release at the tip of neurites of differentiated neurons through direct binding to the presynaptic protein synaptotagmin I [64].

Other mechanisms modulating the activity of the presynaptic terminal involve the function of cell adhesion proteins, most notably trans-synaptic cadherin interactions. It is widely accepted that cadherin-*β*-catenin adhesion complexes have an essential function during the recruitment and clustering of SVs to synapses [65–69]. Indeed, ablation of *β*-catenin results in the mislocalization of SVs along the axon, while clustering of active zone proteins like Bassoon is unchanged [68]. Tyrosine 654 phosphorylation of *β*-catenin weakens cadherin-catenin interactions [70]. Interestingly, the tyrosine kinase FER, which is an ASDs' candidate gene [71], activates the tyrosine phosphatase SHP-2 which removes *β*-catenin phosphorylation and strengthens cadherin mediated adhesion [72]. Among other proteins modulating *β*-catenin dissociation from cell adhesion complexes that have been genetically linked with ASD is the MET receptor tyrosine kinase [30], which phosphorylates Tyr142 in *β*-catenin and promotes its dissociation from cadherins [73], thus linking regulation of cell adhesion by catenins in the pathophysiology of ASDs. In sum, the data available indicates an essential role for Wnt/*β*-catenin signaling in synaptic structure stability and function through modulating cell adhesion, vesicle exocytosis, and clustering well beyond *β*-catenin functioning solely as a TCF/LEF transcriptional coactivator.

## 4. ASDs and Wnt Signaling at the Postsynaptic Terminal

Experience driven plasticity is highly dependent on proper synaptic transmission and is mainly modulated by Ca^2+^ related pathways. Canonical and noncanonical Wnt pathways have been extensively related to Ca^2+^ homeostasis and signaling [47, 54, 74, 75]. Ligands such as Wnt3a [54], Wnt5a [75], and Wnt7a [47] have all been shown to increase Ca^2+^ influxes in neurons. It is accepted that activation of L-type voltage sensitive Ca^2+^ channels (L-VSCCs) or NMDA receptors allows the entrance of Ca^2+^ which in turn activate CAMKII triggering actin cytoskeleton reorganization to regulate dendritic growth [76]. In this regard, CAMKII and the Wnt target gene CAMKIV [77] activate transcription factors such as CREB to start activity dependent transcription to further promote synaptic development [78]. CAMKIV has been associated with ASD [79] and additionally it mediates *β*-catenin dependent dendritic growth upon Ca^2+^ influx [78, 80].

Activation of CAMKII and other kinases through NMDAR-mediated Ca^2+^ influx is an event preceding the establishment of long-term synaptic potentiation (LTP) that allows the recruitment of AMPARs at the postsynaptic terminal, which in turn enhances long lasting excitatory transmission [81]. Additionally, CAMKII robustly phosphorylates the cell adhesion neuroligin 1 (*NLGN1*) protein increasing its surface expression [82]. Notably, suppression of Wnt/*β*-catenin signaling impairs LTP and conversely its activation facilitates it [53], and both enhanced and diminished LTP have been observed in animal models of ASD. For instance, given that enhanced LTP has been observed in TSC2 mutant model [83] and that* TSC2* missense mutations fail to inhibit the Wnt pathway [26], it is likely that overactivation of the signaling cascade may enhance LTP in this specific model. In contrast, mutant models for Fragile X mental retardation-1 (*FMR1*), and also for the disrupted in schizophrenia 1 (*DISC1*) genes, exhibit diminished capacity to establish LTP [84, 85]. Besides their putative role in schizophrenia, ASDs and other neurological diseases [86–88], common* DISC1* genetic variants, directly impact Wnt/*β*-catenin signaling function (see below) [89]. Altogether, the data suggest that the Wnt/*β*-catenin pathway plays a central role in Ca^2+^ homeostasis at postsynaptic terminals, which is commonly disrupted in ASD. In addition, abnormal establishment of LTP, phenomenon in which the signaling cascade plays an important role, has profound effects in activity driven plasticity affecting efficient synaptic transmission and disrupting the E/I balance.

LTP is the most well understood paradigm of activity driven plasticity and is considered to be one of the synaptic mechanisms underlying learning and memory [81]. In turn, several aspects of the ASD core symptomatology and the high comorbidity with intellectual disability disorder could be explained by defective memory mechanisms [90]. Indeed, diminished episodic memory has been reported for high functioning ASD individuals and is thought to impair the relational binding of elements comprising complex stimuli [91]. Therefore, rescuing defects in LTP that appears to be highly regulated by the Wnt/*β*-catenin pathway specifically through the modulation of GSK3*β* could improve core ASD symptomatology and open a therapeutic window for the treatment of ASD through the fine-tuning of synaptic plasticity.

## 5. Synaptic Wnt/GSK3***β*** Signaling Hub in ASD

GSK3 is an evolutionary conserved serine/threonine kinase highly abundant in the brain. Two homologous isoforms, GSK3*α* and GSK3*β*, have been described in mammals and are involved in multiple cellular processes including glycogen metabolism, gene transcription, microtubule stability, and apoptosis [92]. GSK3*β* is as a convergence point of major prevalent neurological disorders, including Alzheimer's disease, schizophrenia, and bipolar disorder [93–95], and its activity is negatively regulated by Wnt signaling. As mentioned before, the* DISC1* gene has an essential role in modulating brain structure and function and when mutated leads to neuropsychiatric behavior. DISC1 inhibits GSK3*β* activity by direct physical interaction resulting in reduced *β*-catenin phosphorylation and activation of Wnt/*β*-catenin signaling cascade [96] and common genetic variants affecting the coding sequence of the gene were found to suppress Wnt/*β*-catenin signaling activity [89]. Regarding ASDs, hyperactivation of GSK3*β* has been documented in animal models of FXS [97–99]. For instance, knock in mice expressing constitutively active form of GSK3*β* displays similar social preference abnormalities as FMR1 KO mice [99].

Mouse models for Fragile X, Phellan-McDermid, and Angelman syndromes, as well as for Tuberous Sclerosis, all present an abnormal number of dendritic spines that suggest a dysregulation in synaptic turnover [100–102]. In this regard, postnatal ablation of GSK3*β* in mice forebrain has anxiolytic and prosocial effects [103] and its overexpression accounts for spatial learning deficits in the Morris water maze paradigm [104]. Interestingly, forebrain deletion of GSK3*β* leads to reduced spine density where persistent spines are lost and newly formed spines are unstable [105]. These structural abnormalities are accompanied by a drop in AMPA dependent mEPSC and the effect is mimicked by the expression of constitutively active *β*-catenin [105]. Furthermore, pharmacological inhibition of GSK3*β* has been shown to increase internalization of NMDA and AMPA receptors, effect that is mainly observed for NR2B containing receptors [106]. Conversely, activation of GSK3*β* impairs the establishment of LTP [107] and high frequency stimulation inhibits GSK3*β* in a Ca^2+^ dependent mechanism [108]. Given that increased abnormal spine density is a pathological hallmark in ASD that may lead to brain hyperconnectivity underlying the basis for E/I balance, the data suggests that inhibition of the Wnt/*β*-catenin signaling through hyperactivation of GSK3*β* might help to explain transmission anomalies as it is observed in ASD.

## 6. Pharmacological Regulation of GSK3***β*** in ASD

Due to its high heterogeneity, genetic factors cannot be held accountable for the entire spectrum of autism suggesting a role for environmental factors in the onset of ASD.* In utero* exposure to anticonvulsive medication is known to cause neurodevelopmental abnormalities [109]. The most well studied anticonvulsive agent in these subjects is valproic acid (valproate, VPA), a known inhibitor of GSK3*β* [110] and of histone deacetylase (HDAC) [111] activities. As an inhibitor of GSK3*β*, VPA induces the stabilization of *β*-catenin and the activation of Wnt target genes, though the exact mechanism of GSK3*β* is not currently understood. Indeed,* in utero* exposure to VPA increases the incidence of autism in the offspring [112, 113] and mice models, which have been prenatally exposed to VPA exhibiting ASD-like behaviors and morphological brain abnormalities [112, 114]. Currently, mice prenatally exposed to VPA (VPA mice) are widely used as animal models to understand the onset/development of ASDs [115]. This VPA mouse model results from intraperitoneal injection in embryonic stages E12–E17, which is a critical period in forebrain development, where dysregulation of Wnt signaling (different time points) induces morphological abnormalities in the brain [116].

While several molecular mechanisms regarding the onset of ASDs in VPA mice have been reported, the activation of Wnt/*β*-catenin signaling is central through the regulation of GSK3*β*. VPA mice exhibit elevated NMDA receptor levels and enhanced LTP [117] and inhibition of GABA transporter VGAT expression in cortical cultures [118], suggesting an important enhancement in excitatory neurotransmission. Likewise, VPA mice induce demethylation of* WNT1* and* WNT2* genes further enhancing Wnt/*β*-catenin signaling [119]. In this regard, sulindac treatment, an anti-inflammatory drug that downregulates Wnt/*β*-catenin signaling by enhancing GSK3*β* expression in the prefrontal cortex or the hippocampal region of VPA mice [120], improved repetitive stereotypic activity, learning and memory, as well as behavioral abnormalities [120, 121]. Interestingly, the VPA transcriptome revealed enhanced expression of multiple genes involved in Wnt/*β*-catenin, neurotrophin, and LTP signaling, the same pathways which also appear enriched in the transcriptome of lithium [122], which mimics Wnt/*β*-catenin signaling by inhibiting GSK3*β* [123]. Nonetheless, although prenatal treatment with VPA appears to enhance the expression of Wnt/*β*-catenin signaling, most of the data comes from* in vitro *cell cultures exposed to VPA and not from* in vivo* studies using mice prenatally exposed to the drug. In this context, it is interesting to note that chronic VPA treatment in mice has been shown to correct dendritic spine deficits and to improve novel object recognition [124]; thus, the postnatal basal activity of the Wnt/*β*-catenin pathway is still unknown. In this context, it is interesting to note that ASD could result from a transient gain of function of the Wnt/*β*-catenin pathway during embryonic development and a subsequent decline after birth.

Lithium has been widely used to manage mood disorders, such as bipolar disorders, and it is not uncommon for ASD children to feature symptoms within this spectrum such as euphoria, mania, or paranoia [125]. Few studies have documented the effects of lithium in ASDs but overall they show promising results as a therapeutic agent. For instance, lithium administration to 30 children and adolescents diagnosed with ASD through DSM-IV-TR criteria improved the symptomatology on 43% of patients [125]. Likewise, chronic administration of lithium to neonatal rats who exhibit ASD-like behaviors abolished their symptoms and improved defects in neurogenesis and E/I balance [126]. Additionally, chronic lithium treatment reversed the increase in cerebral protein synthesis and ameliorates behavioral abnormalities commonly observed in FXS mice models [127], probably through inhibitory GSK3*β* phosphorylation (phosphor-Ser9 and phosphor-Ser21) [128]. Interestingly, pharmacological inhibition of GSK3*β* rescues LTP and hippocampal neurogenesis defects in FMR1 knockout mice and improves cognitive tasks [97, 103]. Furthermore, GSK3*β* inhibition similarly rescues dendritic spines deficit observed in FXS mice suggesting that inhibition of this kinase and thus activation the Wnt/*β*-catenin play a role in reactivating synaptic plasticity and these effects might play an important role in the behavioral and learning improvements observed.

Antagonists for metabotropic glutamate receptor (mGluRs) are up to date the most successful pharmacological modulators improving ASD symptomatology probably through regulation of abnormal mRNA translation at synapses [129]. In this context, the use of MPEP (2-methyl-6-phenylethynyl-pyridine), mGluR5 antagonists, increases inhibitory GSK3*β* phosphorylation selectively in* FMR1* knockout mice [130], effect that is mimicked by chronic lithium treatment. Moreover, this compound corrects dendritic spine deficits through upregulation of PSD-95 and learning impairment in FXS mice model [131], further ascribing a regulatory function directly at the synapses as the underlying mechanism for the therapeutic effect. Finally, MPEP treatment induces the expression of several pathways, including those governed by Wnt signaling in the frontal cortex of rats [132].

## 7. Concluding Remarks

ASD displays a high genetic heterogeneity that results in a wide range of abnormal phenotypes and settling a unified paradigm that accounts for the gain or loss of function of genetically associated genes has been an elusive task. Currently, most elements associated with ASDs converge in signaling pathways important for synaptic plasticity, where Wnt/*β*-catenin signaling plays a central role. As described in this review, several lines of evidence indicate that Wnt signaling regulation of serine/threonine kinase GSK3*β* has profound effects in activity dependent synaptic plasticity and thus in the regulation of the E/I balance. Through dissecting Wnt/GSK3*β* activity and pharmacology in cells and animal models of ASDs, it seems plausible that there may be differential effects driven by Wnt/*β*-catenin signaling activity during the initial patterning of brain structures and later on when these structures have been established. Overall, the therapeutic value of GSK3*β* modulation that seems to rescue synaptic plasticity events that could be disrupted in ASD brains warrants further basic and clinical investigation.
